# RNA Sequencing Reveals Widespread Transcription of Natural Antisense RNAs in *Entamoeba* Species

**DOI:** 10.3390/microorganisms10020396

**Published:** 2022-02-08

**Authors:** Damien Mornico, Chung-Chau Hon, Mikael Koutero, Christian Weber, Jean-Yves Coppée, C Graham Clark, Marie-Agnes Dillies, Nancy Guillen

**Affiliations:** 1 Institut Pasteur, Université de Paris, Bioinformatics and Biostatistics Hub, 75015 Paris, France; marie-agnes.dillies@pasteur.fr; 2 Institut Pasteur, Unité Biologie Cellulaire du Parasitisme, 75015 Paris, France; christian.weber@pasteur.fr; 3 Institut National de la Santé et de la Recherche Médicale, INSERM U786, 75015 Paris, France; 4 Institut Pasteur, Plate-Forme Transcriptome et Epigénome, 75015 Paris, France; mikael@koutero.me (M.K.); jean-yves.coppee@pasteur.fr (J.-Y.C.); 5 London School of Hygiene and Tropical Medicine, London WC1E 7HT, UK; graham.clark@lshtm.ac.uk; 6 Centre National de la Recherche Scientifique, CNRS ERL9195, 75015 Paris, France

**Keywords:** parasite, genomics, transcriptomics, antisense RNA

## Abstract

*Entamoeba* is a genus of Amoebozoa that includes the intestine-colonizing pathogenic species *Entamoeba histolytica*. To understand the basis of gene regulation in *E. histolytica* from an evolutionary perspective, we have profiled the transcriptomes of its closely related species *E. dispar*, *E. moshkovskii* and *E. invadens.* Genome-wide identification of transcription start sites (TSS) and polyadenylation sites (PAS) revealed the similarities and differences of their gene regulatory sequences. In particular, we found the widespread initiation of antisense transcription from within the gene coding sequences is a common feature among all *Entamoeba* species. Interestingly, we observed the enrichment of antisense transcription in genes involved in several processes that are common to species infecting the human intestine, e.g., the metabolism of phospholipids. These results suggest a potentially conserved and compact gene regulatory system in *Entamoeba*.

## 1. Introduction

*Entamoeba* organisms are endobiotic amoebae colonizing species of animals. The persistence of at least seven species of *Entamoeba* depends on their ability to infect humans, mainly in the intestinal tract, where they divide and encyst [1]. There has been a renewed interest in commensal intestinal amoebae because, being members of the human eukaryome, these microorganisms can be an important component in the establishment and functioning of intestinal homeostasis [2,3]. One of these species is *E. histolytica*, the etiological agent of amebiasis. As a pathogenic parasite, *E. histolytica* varies between the commensal status seen in 90% of infected people and the virulent status that leads to intestinal invasion in the remaining 10% [4]. Other species of *Entamoeba* are intestinal commensals and include *E. dispar* which has a life cycle similar to that of *E. histolytica* [1]. *E. histolytica* and *E. dispar* together account for nearly 82% of *Entamoeba* infections in humans. Infections with *E. dispar* seems to be ~10 times more common than *E. histolytica* [5]. The remaining *Entamoeba* infections correspond to other non-pathogenic species, *E. coli* (1.98%), *E. hartmanni* (0.96%), *E. polecki* (0.04%) and *E. gingivalis* (4.6%) [5]. Another species related to *E. histolytica* is *E. moshkovskii*, a free-living amoeba commonly found in sewage and known to infect humans, although its pathogenicity is not precisely determined [6,7]. Overall, most *Entamoeba* infections in humans seems to be caused by commensal species and these species feed on and evolve with *Enterobacteriaceae*. Another widely studied species is *E. invadens*, which is pathogenic in a wide range of reptiles [8,9]. *E. invadens* is used as a model for the studying the formation of amoebic cysts, due to the possibility of completing its cell cycle under laboratory conditions.

The genomes of *E.* *histolytica* strain HM1:IMSS [10,11,12]; *E. dispar* strain SAW760 [13], *E. moshkovskii* strain Laredo [14] and *E. invadens* strain IP-1 [15,16] have been sequenced. All these genomes are AT-rich, although *E. invadens* and *E. moshkovskii* genomes have unusually biased GC distributions [14]. *E. histolytica* and *E. dispar* are closely related species, bearing 95% and 85% of nucleotide identity in their genic and intergenic regions, respectively [17,18]. The genome of *E. moshkovskii* is slightly larger than that of *E. histolytica*, and the phylogenetic relationship between diverse isolates of *E. moshkovskii* indicates that their most recent common ancestor is 500 times more ancient than the ancestor of isolates from *E. histolytica* [14]. The parasite of reptiles, *E. invadens*, is readily distinguishable from the other human infecting species, sharing only 74% and 62% of nucleotide identity in their genic and intergenic regions, respectively [15,16]. Despite transposable elements being abundant in all four genomes [19,20], there are clear distinctions among species: While DNA transposons dominate in *E. moshkovskii* and *E. invadens*, retrotransposons dominate in *E. histolytica* and *E. dispar* [21]. These transposons are often located near genes encoding tRNA, which are organized in tandem arrays [22,23]. *E. histolytica* has the most compact genome among the four species, with ~8300 coding genes in 20 Mb, compared to the *E. invadens* genome which bears 11,549 coding genes in 40 Mb [14]. The gene lengths of *E. histolytica* and *E. invadens* are similar while the intergenic regions in *E. invadens* are longer than those in *E. histolytica* (408 bp vs. 282 bp) [16]. A total of 4704 gene families comprising 21,741 genes are shared among all four species [14].

Transcriptomic analyses enabled the identification of gene promoter sequences in *E. invadens* and *E. histolytica* [24,25,26,27,28], as well as the characterization of small RNAs regulating encystation of *E. invadens* and gene silencing in *E. histolytica* [29,30,31]. More recent analyses using RNA sequencing (RNA-Seq), essentially performed in *E. histolytica*, enabled genome-wide identification of transcription start sites (TSS) [32] and polyadenylation (polyA) sites (PAS) [33,34], as well as regulatory RNA such as miRNAs [35], small RNAs [29] and long non-coding RNAs [36]. More recently, natural antisense RNAs (NATs) [32], which are non-coding RNAs transcribed from the opposite strand of a gene, have been described in *E. histolytica*. NAT transcription is abundant as roughly 25% of genes generate NATs in all environmental conditions tested [32]. In addition, genomic sequences for TSS and PAS are similar for sense and antisense transcription, which indicates a novel transcriptional regulatory scenario probably derived from the compactness of the *E. histolytica* genome [32]. These characteristics of RNA biogenesis in the related species *E. dispar*, *E. moshkovskii* and *E. invadens* have not yet been completely described.

In this study, we aimed to study the conservation of gene regulatory sequences (i.e., motifs at TSS and PAS) and NAT transcription between *E. histolytica*, *E. dispar*, *E. moshkovskii* and *E. invadens*. We analysed similarities and differences in transcriptional genomic determinants for mRNA and NATs and concluded that TSS for NATs occurs on the opposite strand within the coding sequence, although variations exist between the species. NATs precisely initiate at the stop codon in *E. histolytica* and *E. dispar* but appear to be dispersed inside the intergenic spacers in the two other species. Comparing the transcriptomic profiles of genes targeted by NATs in *E. histolytica* to *E. dispar* and *E. moshkovskii*, we discovered significant biological processes common to amoebic species infecting the human intestine, with lipid metabolism activities as the most important enriched functions, whereas the comparison with *E. invadens* highlighted vesicular trafficking and regulation of RNA transcription. Common gene products and functions associated with NATs identify robust candidates for monitoring NAT biogenesis in all four species of Entamoebidae.

## 2. Materials and Methods

### 2.1. Entamoeba Species and In Vitro Culture

*Entamoeba histolytica* strain HM-1: IMSS was isolated in 1967 from a biopsy of a rectal ulcer from an adult male with amoebic dysentery, Mexico City, Mexico. *Entamoeba dispar* strain SAW760 was isolated in 1979 from an asymptomatic adult male at the London School of Hygiene and Tropical Medicine, UK. The Laredo strain of *Entamoeba moshkovskii* was isolated from a resident of Laredo, TX, USA, who showed symptoms of diarrhoea. *Entamoeba invadens* strain IP-1 was isolated in Canada from a natural infection of a painted turtle *Chrysemys picta*. The HM-1: IMSS strain was a gift from Professor Ruy Perez Tamayo and Dr Alfonso Olivos (UNAM, Mexico). The three other strains are from the collection of Professor Graham Clark. All the strains were cultured in axenic media whose compositions have been described previously [37]. *E. histolytica* strain HM-1: IMSS was cultured in TYI-S-33 medium at 37 °C; *E. moshkovskii* strain Laredo was cultured in LYI-S-2 medium at room temperature; *E. dispar* strain SAW760 was cultured in LYI-S-2 medium at 37 °C and *E. invadens* strain IP-1 was cultured in TYI-S-33 medium at 25 °C.

### 2.2. RNA Preparation

Total RNA was extracted from approximately 1 × 10^6^ trophozoites (with each culture performed in triplicate) using Trizol (Invitrogen, Thermo Fisher Scientific, Waltham, MA, USA, ref. 15596026). The poly(A) fraction was purified from 10 to 100 µg of total RNA using Dynabeads according to the manufacturer’s instructions (Thermo Fisher Scientific ref. 28152103011150). Small RNAs were purified from 10 μg of total RNA loaded on a denaturing 15% TBE- Urea gel (BioRad, Marnes-La-Coquette, France, ref. 456-6053). More specifically, following ZR-small-RNA Ladder (Zymo Research, Tustin, CA, USA, ref. R1090) size indications, a fragment of gel corresponding to the 15–35 nt region was excised with a scalpel and RNAs were extracted using the ZR small-RNA PAGE Recovery Kit (Zymo Research, ref. R1070) following manufacturer’s recommendations. Small RNAs were then treated with Tobacco Acid Pyrophosphatase (Epicentre Biotechnologies, Madison, WI, USA, ref. T19100) for 1 h and purified by RNA Clean and Concentrator-5 (Zymo Research, ref. R1015) following the manufacturer’s instructions.

### 2.3. Library Construction, Sequencing and Read Processing

First, libraries were constructed using TruSeq Small RNA Sample Prep Kit (Illumina, San Diego, CA, USA, ref. RS-200-0012) following the manufacturer’s instructions. Then, all the libraries were purified on a 5% TBE gel (BioRad, ref. 456-5013) and were quantified by Bioanalyzer DNA High Sensitivity Chips (Agilent Technologies, Santa Clara, CA, USA, ref. 5065-4626). Sequencing was performed on HiSeq-2000 (Illumina) in a multiplexed 51 + 7 nucleotide single-end read using a TruSeq SR Cluster kit v3 cBot HS (Illumina, ref. GD-401-3002) and a TruSeq SBS kit v3 HS 50 cycles (Illumina, ref. FC-401-3002). Finally, FASTQ sequence files were generated using CASAVA 1.8.2 (Illumina) and adapter sequences were trimmed using AlienTrimmer [38] (v. 0.4.0). Reads shorter than 20 nucleotides were discarded.

### 2.4. Reference Genomes

The reference genome sequences and annotations of *E. histolytica* (20.80 Mb, 1496 contigs and 8201 genes), *E. dispar* (22.96 Mb, 3312 contigs and 8744 genes), *E. moshkovskii* (25.25 Mb, 1147 contigs and 12,260 genes) and *E. invadens* (40.88 Mb, 1144 contigs and 11,997 genes) were downloaded from AmoebaDB v46 (https://www.amoebadb.org/common/downloads/release-46, accessed on 5 January 2022).

### 2.5. Data Repositories

The data are available in the SRA database (https://www.ncbi.nlm.nih.gov/sra/) under accession number PRJNA781395.

### 2.6. De Novo Assembly of Transcriptomes

Libraries within each species (Table S1) were merged for mapping and de novo assembly. First, reads were mapped to the corresponding reference genomes of each species using STAR v2.5.0 [39] with a maximum intron length of 900 (—alignIntronMax), maximum mismatches of 2 (—outFilterMismatchNmax), maximum multi-mapping locations of 10 (—outFilterMultimapNmax) and minimum overhang of 25 (—alignSJoverhangMin) for spliced alignments. Mapped reads were split into plus and minus strand-sets with Samtools v. 1.13 [40]. Then, de novo assembly of transcript fragments (i.e., contigs) were performed on each strand-set using Trinity [41], with a kmer size of 15 nt (—KMER_SIZE 15), a maximum intron size of 900 nt (—genome_guided_max_intron 900), a minimum coverage of 20 (—genome_guided_min_coverage 20) and a minimum length of 150 nt (—min_contig_length 100). Lastly, resulting transcript contigs were mapped on the corresponding reference genome using Gmap [42] with a maximum intron length of 900 nt (-K 900). Transcript contigs mapped to the opposite strands of coding genes (with a minimum 10% contig length, detected using feature Counts software [43] with —fracOverlap 0.1) were defined as “NAT fragments”. Genes were defined as “NAT genes”, when at least 1 NAT fragment mapped. In order to specify parts of genes preferentially covered by NAT fragments, we split the genes in 5 equal regions and counting was performed on each region as well.

### 2.7. Analyses of Small RNAs

The small RNA data were processed as previously described [44]. Briefly, deduplicated reads were removed using fqCleanER (https://gitlab.pasteur.fr/GIPhy/fqCleanER, accessed on 10 August 2021). The remaining unique reads were then mapped to the corresponding reference genomes using Bowtie v. 0.12.7 [45] with maximum 1 end-to-end mismatch (-v 1) and all mapped locations were reported (—all). Genes with ≥20 small RNA reads were defined as sRNA targeted genes.

### 2.8. TSS Identification and Annotation

The reads were aligned to the different reference genomes, using Bowtie v. 0.12.7 [45] with the following parameters: maximum of 2 mismatches were allowed (-*n* 2) and reads mapped to multiple locations (-m 50) were reported only once (-k 1). The alignments produced were sorted and indexed with SAMTools [40]. Coverage graphs representing the numbers of mapped reads per nucleotide were generated based on the sorted reads using BEDTools [40,46], focusing on 5′ end position (−5). On each coverage, an upper quartile normalization [47] was performed and a minimum coverage of 4 was imposed. TSS candidates within 10 nts from each other were then clustered together in transcription initiation clusters and the position of the strongest coverage was defined as the peak. Each TSS was then classified as a gene TSS (gTSS), an internal TSS (iTSS), an antisense TSS (asTSS), or an orphan (oTSS) if it could not be assigned to any of the previous classes [48]. A TSS was classified as a gTSS if it was located ≤100 bp upstream of a gene and as an asTSS if it was located within the 200 bp surrounding the stop codons. The TSS with the strongest expression values (maximum peak height) among gTSS of a gene was classified as primary (pTSS). iTSS were located within an annotated gene on the sense strand.

### 2.9. PAS Identification and Annotation

First, reads with a stretch of five or more ‘A’s at their ends (or ‘T’s at their beginning) were selected for this analysis, as they potentially contain mRNA poly(A) tails. Redundant reads were removed and stretches of As at the ends were trimmed. Remaining reads with a minimum length of 18 nt were then mapped on the reference genome using Bowtie [45] with following parameters: -*n* 2 -k 1 -m 50 -l 30. To avoid false positives due to sequencing errors, reads with low quality (<20) around PAS (5 nt upstream and downstream) were removed from the set. To discriminate real poly(A) tracks of true polyadenylation from poly(A) tracks of internal homopolymeric stretches on the mRNAs, false positives were discarded if they met the following criteria: (i) reads with ≥8 nt within 10 nt immediately upstream of the PAS are A’s, (ii) mapping with ≥5 ‘A’s immediately downstream of the PAS.

PAS candidates within 12 nts from each other were then clustered together in PAS clusters and the position of the strongest coverage was defined as the peak. PAS with fewer than 2 reads of coverage at the peak were removed.

### 2.10. Motif Enrichment

The sequences immediately upstream and downstream of the gTSS, and asTSS (100 nt on each side), as well as the PAS of mRNA and NAT were used to scan for conserved motifs using DREME [49]. The immediate upstream or downstream sequences were thus used as the positive sets, and the farther upstream (at position −200) or downstream (at position +150) sequences of the same length were used as the negative sets. To visually investigate the positional enrichment of these discovered motifs surrounding the polyadenylation sites, the total occurrence of these motifs was searched along the sequences surrounding (300 nt) the poly(A) sites.

### 2.11. Orthologs, Core and Pan-Genome Identification

The four species’ proteomes were used to compute orthologous groups of genes among all strains with OrthoFinder v. 2.3.8 [50], with the Blast sequence comparison option (-S blast) and default MCL inflation parameter (-I 1.5). Orthologous groups with genes present in each species as a unique copy were identified as “core-genome”. Genes involved in synteny groups have been identified in AmoebaDB (https://amoebadb.org, accessed on 5 January 2022).

### 2.12. Differential Expression Analysis

Firstly, reads of each replicate were mapped to the reference genome of *E. histolytica*, using STAR v. 2.5.0 [39]) with a maximum intron length of 900 (—alignIntronMax), 2 mismatches maximum (—outFilterMismatchNmax), 10 locations maximum for a read mapping (—outFilterMultimapNmax), minimum overhang of 25 nt for spliced alignments. Secondly, read counts for each gene in each sample were computed with featureCounts [43] separately on the direct (-s 1) and opposite strand (-s 2), allowing multi-mapping reads (-M). In order to take into account length differences between orthologous genes across species, we first applied a Transcript Per Million (TPM) quantification [51]. The DESeq2 normalization was used: size factors were computed on sense counts and were then applied to both sense and antisense counting. Finally, transcript differential expressions were calculated on the merged normalized counts (sense and antisense) using DESeq2 v. 1.24.0 [52] within the SARTools pipeline v. 1.7.2 [53]. False discovery rates were corrected using the Benjamini–Hochberg procedure. The analysis was conducted in R [54] and figures were produced using the ggplot2 package [55].

### 2.13. eggNOG Annotations and GO Terms Enrichment

To combine gene datasets the Venn diagram approach was used (https://bioinfogp.cnb.csic.es/tools/venny/, accessed on 15 December 2021). Predicted proteins were retrieved using UniProt (https://www.uniprot.org/, accessed on 15 December 2021) using gene name and the Uniprot function. For annotation, predicted proteins of *Entamoeba* were assigned to the orthologous groups of the EggNOG database v. 5 [56] using the eggNOG-mapper. V. 2 (http://eggnog-mapper.embl.de/, accessed on 27 August 2021) according to their defined parameters (hit e-values ≤ 1e-3). One-letter abbreviations for the functional categories’ correspondence are at https://www.ncbi.nlm.nih.gov/research/cog#, accessed on 27 August 2021. Further identification of protein classes and gene ontology enrichments corresponding to genes harbouring sense and antisense transcripts were performed with Amoeba DB tools: search for gene ID, data analysis for GO terms (biological processes and molecular functions) and synteny search in the setting pre-configured table, orthologs and paralogs within VEuPathDB (https://amoebadb.org/amoeba/app, accessed on 28 December 2021). For proteins related to lipid metabolism analysis we used UniProt (https://www.uniprot.org/, accessed on 28 December 2021) and STRING (https://string-db.org/ accessed on 28 December 2021) clustering methods.

## 3. Results

### 3.1. Widespread NAT in Entamoeba Species

**Figure 1:**
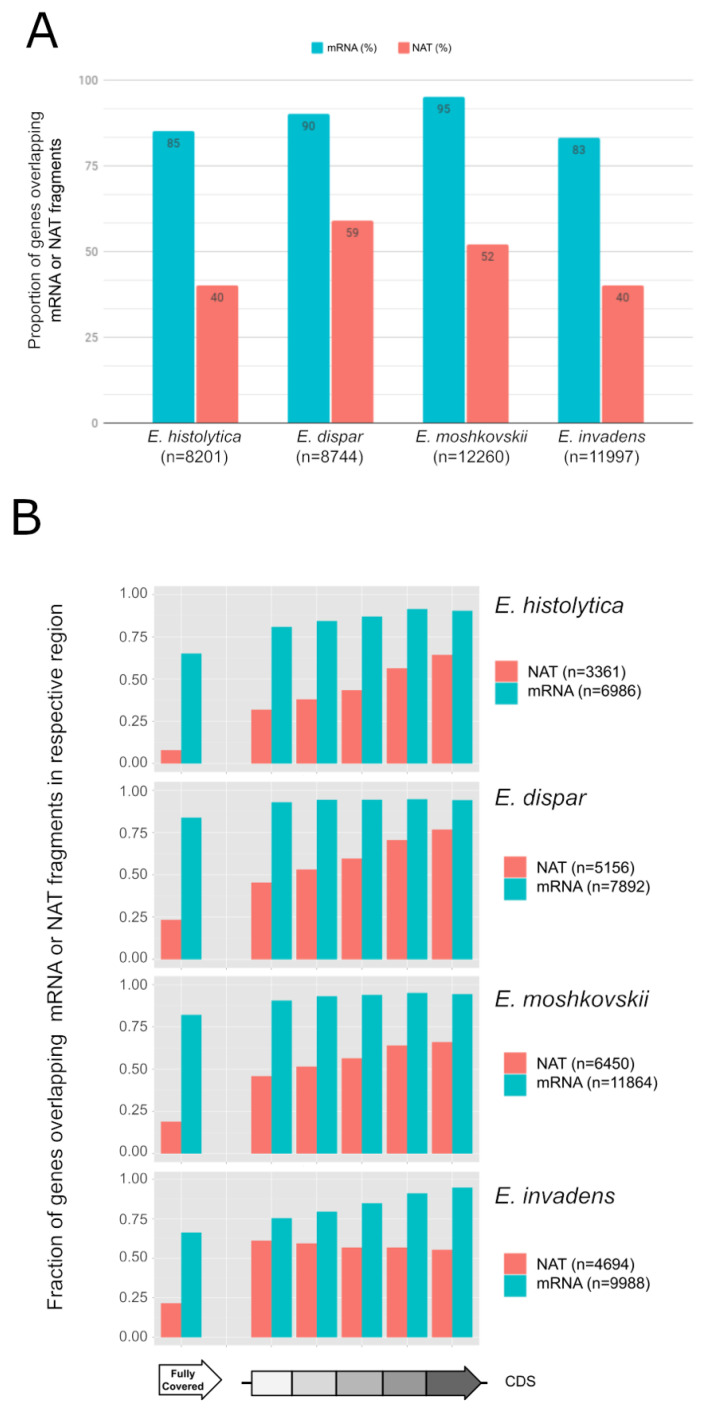
NATs and mRNA among the species. (**A**) Proportion of CDSs linked to mRNAs (blue) and NATs (red) in the 4 species. (**B**) Proportion of genes overlapping at least one mRNA (blue) or NAT (red) contig in the 5 different regions of their CDS on both sides in each species.

To compare the transcriptomes between the four species of *Entamoeba*, RNA-seq was performed on polyadenylated RNAs (Table S1). De novo assembly of the RNA-seq data yielded 35,000 to 57,000 transcript fragments (i.e., contigs), with mean lengths of 251 to 403 nt, among the four species (Table S2). In total, 15,624 to 27,687 of these contigs can be mapped to the sense strand of genes in their corresponding genome, covering 83% to 95% of genes, attesting to the good representation of the global transcriptome from each species (Figure 1A and Table S3, Sheet 1). From 11% to 27% of these contigs are mapped in intergenic regions and are probably derived from unannotated or non-coding genes (Table S2). Interestingly, 6401 to 13,462 contigs, with mean lengths between 205 nt and 339 nt, can be mapped to the antisense strand of genes, covering 38% to 59% of the genes among the species, suggesting widespread NAT in *Entamoeba* (Figure 1A and Table S3, Sheet 2). Of the four species, *E. dispar* showed the greatest proportion of genes with NAT (~59%). Previous studies have shown that NATs are shorter than the corresponding gene and more likely to map to the 3′ region in *E. histolytica* [32]. After dividing the coding sequences into five equal sized regions, we investigated the coverage pattern of the mRNA and NAT contigs among the four species (Figure 1B). From 65% to 83% of genes are fully covered by mRNA contigs, homogeneously between regions, confirming the good distribution of the transcriptome. The 3′ biased mapping of NAT contigs was present in *E. histolytica*, *E. dispar* and *E. moshkovskii*, while *E. invadens* displayed a uniform pattern of coverage.

### 3.2. Small RNAs and NATs Are Independent Entities of Non-Coding RNAs

Previous studies demonstrated the existence of a distinct class of 27 nt small RNAs in *E. histolytica* and *E. invadens* [29,30,44,57]. Here we sought to investigate whether the biogenesis of NAT and these small RNAs are related or not. We thus purified and sequenced the small RNAs in the four *Entamoeba* species and investigated the correlation of the genome-wide distributions of small RNAs and NAT in each species. As formerly highlighted [44], two main populations of sRNA are identified in *E. histolytica* and *E. invadens*, based on their size distribution (Figure S1A), with two peaks at 27 nt and 31 nt. The same distribution is observed in *E. dispar* and *E. moshkovskii* sRNA populations. Among the four species, ~8% to ~10% of genes were found to overlap antisense small RNAs (Figure S1B and Table S4, Sheet 2), which is much lower than the genes overlapping NATs, described above and only 9 of these genes are common to all the species (according to the orthology analysis described below). In addition, we noticed a bias in the distribution of small RNAs towards the 5′ end of genes in *E. histolytica* and *E. dispar*, and a more spread-out distribution in *E. moshkovskii* and *E. invadens* (Figure S1C), in contrast to the general bias of NAT toward the 3′ end of genes. Then, we used χ^2^ tests in order to further investigate associations between sRNA and NAT distributions (Table S4, Sheet 1). Independence of distributions was rejected in each species. We observed that sRNA and NATs tend to exclude each other. In the genomes we analysed, there was a higher number of NAT genes found in the absence of sRNA (>92%) than when both co-occurred. Reciprocally, a higher number of sRNA genes were found without NATs (from 55% to 71%). Co-occurrence of both NATs and sRNA (in blue) was low compared to expected values (green). We concluded that strong positive association between NATs and sRNA was unlikely. The lack of correlation between the genome-wide distributions of NAT and small RNAs suggests the biogenesis of these two classes of RNAs might be independent.

### 3.3. Identification of TSS for mRNA and NATs

**Figure 2:**
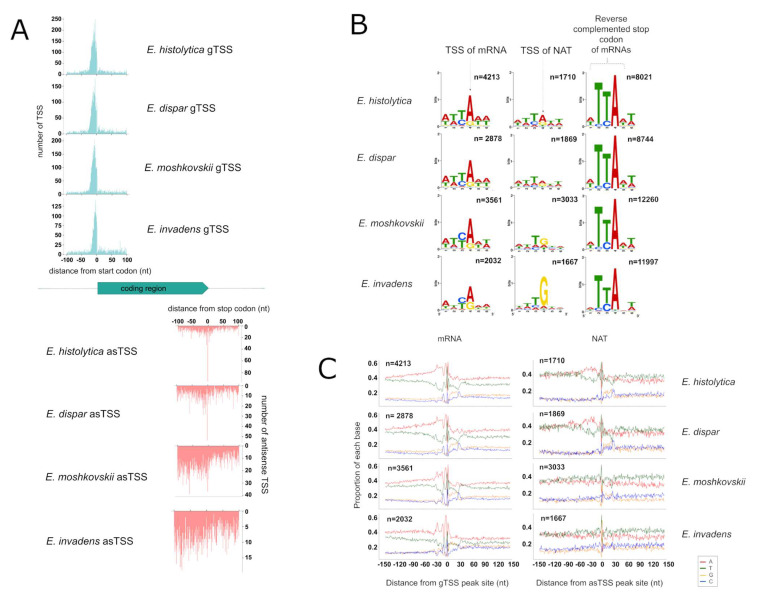
Transcription start sites of mRNA and NATs. (**A**) Mapping of TSS at CDS boundaries (with 100 nt apart) on both strands in the different species. (**B**) Sequences logo computed around stop codon using the entire genome CDS reverse complemented (right), asTSS (middle) and mRNA TSS (left) found in different species. (**C**) Nucleotide sequence composition around TSS of both mRNA and NAT. When several are assigned to a same gene, only the strongest TSS is considered (primary). Notice that 0 corresponds to the TSS peak identified.

We have recently reported that most NATs in *E. histolytica* are initiated from the 3′ end of the coding sequences (CDS), and in particular at the stop codon, which acts as the Initiator (Inr) motif at the TSS of NATs [32]. In this study, we sought to investigate this aspect in other *Entamoeba* species. Using the method previously described [32], we mapped the TSS in the genomes of all four species. Gene TSSs (gTSS), defined as the TSS closest to the start codons (–100 nt) on the sense strand, were identified in *E. histolytica* (*n* = 4213, in 3649 genes), *E. dispar* (*n* = 2878, in 2543 genes), *E. moshkovskii* (*n* = 3561, in 3221 genes) and *E. invadens* (*n* = 2032, in 1756 genes). Antisense TSSs (asTSS), defined as TSSs around the stop codon (+ /−100 nt) on the antisense strand, were also mapped in *E. histolytica* (*n* = 1710, in 1197 genes), *E. dispar* (*n* = 1869, in 969 genes), *E. moshkovskii* (*n* = 3033, in 1162 genes) and *E. invadens* (*n* = 1667, in 639 genes) (Table S5). While gTSS are strongly enriched around 10 nt upstream the start codons, the distribution of asTSS is not as well-defined among the four species (Figure 2A). Indeed, we observed a sharp peak of asTSS at the stop codon in *E. histolytica* and *E. dispar*, while the asTSS in *E. moshkovskii* and *E. invadens* showed a spread distribution at the 3′ end of the CDSs (Figure 2A). These differences could indicate different genomic sequences regulating the initiation of NATs between the two closest species, *E. histolytica* and *E. dispar*, compared to the others (Figure 2B). Indeed, the Inr motifs of gTSS and asTSS seem similar to the stop codon in *E. histolytica* and *E. dispar*; it is the case for gTSS in *E. moshkovskii* and *E. invadens* although bases C and G appeared more often in their gTSS. The tendency to include C and G in Inr motifs from *E. moshkovskii* and *E. invadens* is even more pronounced in the case of asTSS, precluding a clear-cut conclusion from these data concerning PolII preferences in *E. moshkovskii* and *E. invadens*.

Sequence compositions surrounding the TSS of mRNA (primary gTSS) and ta-NAT (primary asTSS) were compared between the four species (Figure 2C). *E. histolytica* and *E. dispar* display clear similarities in nucleotide proportion on both sense and antisense strands, including (i) an A-rich region around −80 to −20 nt, (ii) a T/A enriched region (at −30 nt) within this A-rich region (iii) a C/T enriched region around −10 nt, (iv) a YA motif around TSS (e.g., Inr in Figure 2C), and (v) a C/G enriched region at +25 nt, implying the sequence determinants to initiate mRNA and NAT transcription are essentially the same in these two species. These regions are also visible around *E. moshkovskii* and *E. invadens* gTSSs, although with slightly different proportions, but not clearly defined around asTSSs.

### 3.4. Identification of PAS for mRNAs and NATs

**Figure 3:**
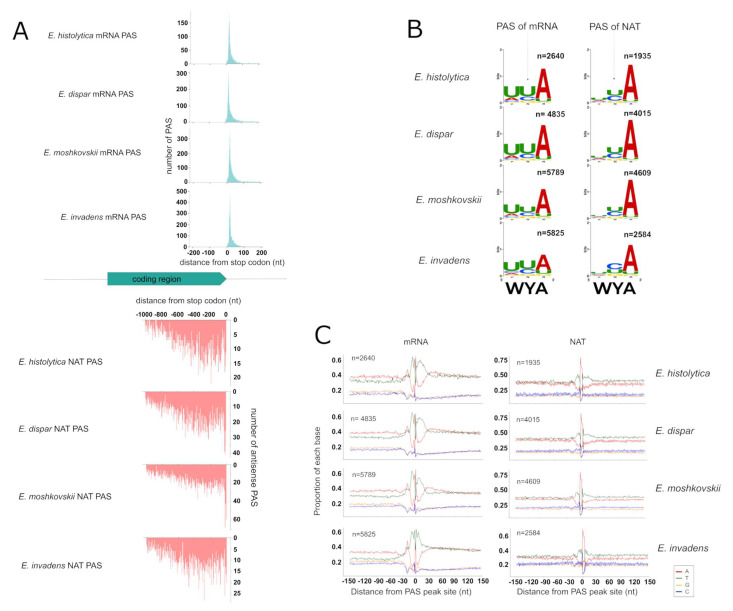
Poly(A) site identification and motif enrichment. (**A**) Distribution of PAS distance from stop codon (position 0) on sense (blue) and antisense DNA strand (red). (**B**) Sequence logo around the PAS of both mRNA (left) and NAT (right). (**C**) Nucleotide sequence composition around the RNA cleavage site (position 0 corresponds to 1 nt before the cleavage site) of both mRNA (left) and NAT (right).

Based on the methods used in our previous study [32], we identified PAS genome-wide in the four species and defined them as mRNA PAS or NATs PAS based on their strands (Table S6). By that definition, 2640 mRNA PAS and 1935 NAT PAS were detected in *E. histolytica*, and similarly, 4835 and 4015 in *E. dispar*; 5789 and 4609 in *E. moshkovskii* and 5825 and 2584 in *E. invadens*. The median distances of mRNA PAS and NAT PAS from the stop-codon are similar in the four species, at 20 to 24 nt and 417 to 494 nt, respectively (Figure 3A). These features are consistent with the previous results [32] and confirm that NATs are mostly shorter than mRNA in *Entamoeba* species.

Analysis of sequence composition around the PAS shows clear similarities among the four species, for both mRNA and NAT (Figure 3B), except for the U-rich region downstream of the PAS (U-rich DSE), which seems shorter in *E. invadens* (from 1 to 15 nt after PAS) than in the others (from 1 to 26 nt after PAS). The cleavage sequence element (CSE), representing the 3 nucleotides around the PAS, is also well identified in all species but slightly less significantly enriched in *E. invadens*. These observations imply that the genomic determinants of polyadenylation are not only conserved between mRNA and NAT, but also conserved among the different species, with slight variations in *E. invadens*, probably reflecting its greater evolutionary distance to the other species.

### 3.5. NAT in the Core-Genome and Species-Specific Genes

**Figure 4:**
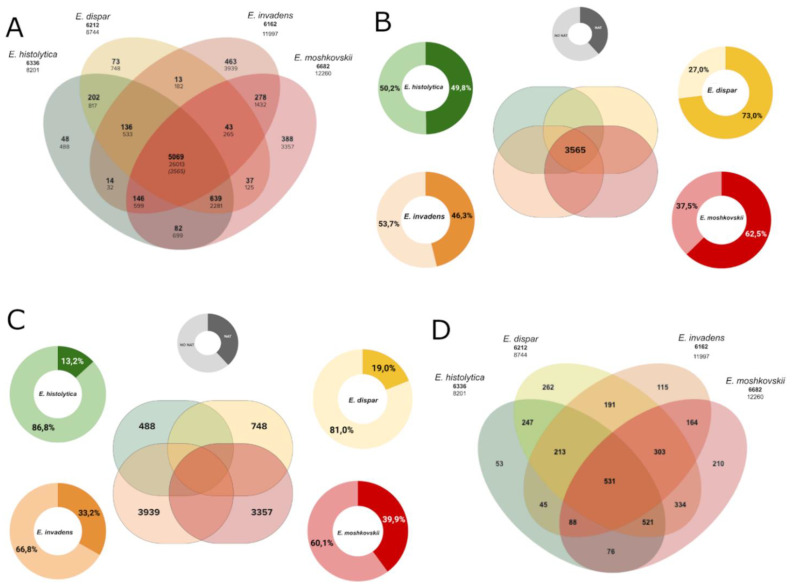
NATs in the core- and pan-genome. (**A**) Venn diagram of specific and orthologous genes among the 4 species. Numbers in bold represent gene families and numbers in regular font represent the genes in those families. The number in italics is the number of families with genes in a unique copy. (**B**) Proportion of NATs in the core-genome for each species. (**C**) Proportion of NAT in specific genes for each species. (**D**) Venn diagram of specific and common NATs in the core-genome.

To investigate the conservation of NATs, we defined groups of orthologous genes among the four species (see Methods). We identified a set of genes that are orthologous among all four species (*n* = 5069), and 3565 of them exist as a unique copy in each species (Figure 4A and Table S3, Sheet 3). Herein we define these 3565 genes as the core-genome, and the genes without an orthologous partner as species-specific genes in each species. Next, we compared the extent of NAT transcription in the core-genome among species. While the orthologs in *E. histolytica* and *E. invadens* showed a similar extent of NAT transcription (~46%), the extent is substantially higher in *E. moshkovskii* and *E. dispar* (65% and 72% respectively) (Figure 4B and Table S3, Sheet 4). Thus, the extent of NAT transcription appears to be higher in the core-genome than in the whole individual genomes, but variations were observed between species.

We also compared the extent of NAT transcription in the species-specific genes. As *E. histolytica* and *E. dispar* have closely related genomes, only a few genes were identified as species-specific (488 and 748, respectively), compared to *E. moshkovskii* and *E. invadens* which harboured large numbers of species-specific genes (3357 and 3939, respectively) (Figure 4A and Table S3, Sheet 7). Among these species-specific genes, *E. histolytica* and *E. dispar* displayed a similar extent of NAT transcription (12% and 19% of genes respectively), compared to the greater extents in *E. invadens* and *E. moshkovskii* (33% and 39%, respectively) (Figure 4C and Table S3, Sheet 8). We noted that these numbers are smaller than the global antisense expression recorded for whole genomes, and variation between genomes is not comparable. *E. dispar*, for instance, showed the smallest extent of NAT transcription in species-specific genes, while it has the largest extent of NAT transcription in the core-genome.

### 3.6. Functional Annotations of NAT-Associated Genes Conserved in All Species

**Figure 5:**
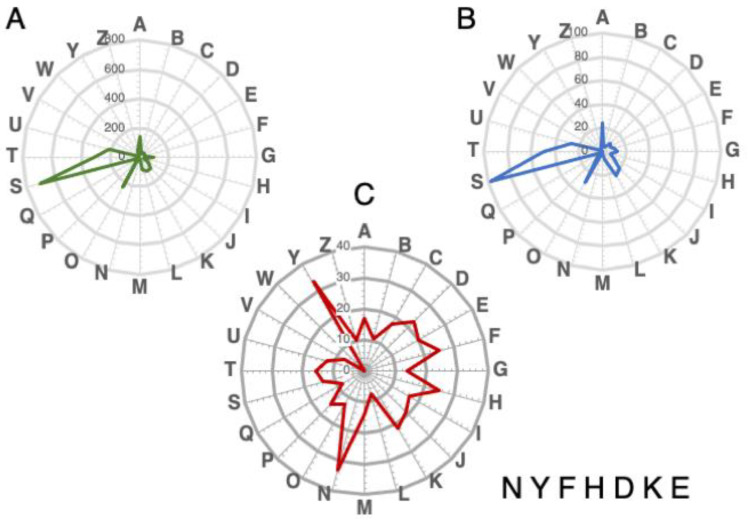
Functional categories of genes targeted by NATs in the core-genome and in the coreNATcore. Predicted proteins of *Entamoeba* from the core-genome and from genes in the coreNATcore were assigned to clusters of orthologous groups of proteins (COG) in the EggNOG database. (**A**) Categories of orthologous groups found in the core-genome. (**B**) Categories of orthologous groups found in the coreNATcore dataset. (**C**) percentage of gene-presenting NATs in relation to the core-genome. One-letter abbreviations for the functional categories correspondence at COG–NCBI (https://www.ncbi.nlm.nih.gov/research/cog, accessed on 27 August 2021) are: A, RNA processing and modification; B, chromatin structure and dynamics; C, energy production and conversion; D, cell division and chromosome partitioning; E, amino acid metabolism and transport; F, nucleotide metabolism and transport; G, carbohydrate metabolism and transport; H, coenzyme metabolism; I, lipid metabolism; J, translation, including ribosome structure and biogenesis; K, transcription; L, replication, recombination and repair; M, cell wall structure and biogenesis and outer membrane; N, motility; O, molecular chaperones and related functions; P, inorganic ion transport and metabolism; Q, secondary metabolites biosynthesis, transport and catabolism; R, general functional prediction only; S, no functional prediction; T, signal transduction; U, intracellular trafficking secretion and vesicular transport; V, defence mechanisms; W, extracellular structures; Y, nuclear structure; Z, cytoskeleton.

About 14% of genes in the core-genome (*n* = 531) are associated with NAT in all four species (hereinafter named the coreNATcore, Figure 4D and Table S3, Sheet 5) and more than half of these (*n* = 271) are annotated as “unknown functions” in AmoebaDB. To investigate the functional enrichment of genes in the coreNATcore, we annotated their potential functions based on orthology mapping using eggNOG-mapper [56] and 360 of them can be mapped to a cluster of orthologous groups (COG) with functional annotations (Table S7, Sheet 1). These include regulation of RNA biosynthesis and transcription (COG categories K, A and J, *n* = 63); signal transduction mechanisms and trafficking (COG categories T and U, *n* = 72); posttranslational modifications, protein turnover and chaperones (COG categories O, *n* = 30); DNA replication, repair, chromatin structure and chromosomes partition (COG categories L, D and B, *n* = 15); transport and metabolism of carbohydrates, lipids, coenzymes, amino acids and ions (COG categories G, F, I, H, E and P, *n* = 46); cytoskeleton (COG categories Z and CZ, *n* = 6); and other COG categories (*n* = 24). In addition, 98 proteins have a recognizable domain but with unknown function (COG category S, *n* = 98). We also annotated the genes in the core-genome using eggNOG-mapper (Table S7, Sheet 2), and 2425 of the 3551 genes can be mapped to a COG. In the core-genome, 20% to 34% of genes within the COG categories N (cell motility), Y (nuclear structure), F (transport and metabolism of nucleotides), H (coenzyme), D (cell cycle), K (transcription), E (amino acids) were associated with NATs (Figure 5) and the COG category W (extracellular structures) was not found in genes associated with NATs. The other mapped functional categories (less than 20%) are summarized in Figure 5.

Previously, we defined a set of NAT-associated genes (*n* = 452) that are common among *E. histolytica* strains under various environmental conditions (defined as *E. histolytica* core NAT) [32], including invasion of the human colon. From those 452 genes, in this work (Table S3, Sheet 5), 98% of them (*n* = 444) are present in that dataset of *E. histolytica* genes presenting NATs. Next, we examined in a Venn diagram the overlap between these *E. histolytica* core NATs and the inter-species coreNATcore defined above and 86 genes were found to be present in both datasets (Table S8, Sheets 1 and 2). In the four species, there is no significant difference between the level of expression of these 86 genes and the level of expression of the other genes according to the comparison test of Wilcoxon (*p*-value = 0.1099, 0.1425, 0.1727 and 0.4273 for *E. histolytica*, *E. dispar*, *E. moshkovskii* and *E. invadens* respectively), (Supplementary Figure S2). They map into 57 contigs (Table S8, Sheet 3) and are not clustered in any particular region within these contigs. Examining the gene ontology (GO) terms of these 86 inter-species common genes (Table S8, Sheet 4), we highlight seven genes involved in metabolism of small molecules, including EHI_000720 which encodes a 5′-methylthioadenosine/S-adenosylhomocysteine (MTA/SAH) nucleosidase. It is involved in the S-adenosyl-L-methionine (SAM, AdoMet) cycle, which recycles S-adenosyl-L-homocysteine back to SAM, and in salvage pathways for 5′-deoxyadenosine and S-methyl-5′-thioadenosine [58]. MTA/SAH nucleosidase plays a key role in the purine salvage pathway and in recycling of methylthio groups, the encoding gene (EHI_000720) is believed to have been acquired by the *E. histolytica* genome through lateral gene transfer (LGT) from a bacterial genome. The six other genes correspond to long-chain-fatty-acid-CoA ligase EHI_029050 (catalysing β-oxidation of fatty acids); triosephosphate isomerase EHI_056480 (enzyme related to glycolysis); galactokinase EHI_094100 (catabolism of galactose); pyridoxal kinase EHI_126090 (phosphotransferase, Vitamin B6 metabolism); enolase EHI_130700 (enzyme related to glycolysis) and leucyl-tRNA synthetase EHI_161970 (ligation of L-leucine to tRNA). Apart from genes with GO term annotations, other proteins involved in known metabolic processes reinforced the above observations. For example, EHI_155520 encodes a protein carrying the C-terminal catalytic domain of glutamine synthetase involved in nitrogen assimilation. Three leucine-rich-repeat-containing proteins (EHI_023340 EHI_024640 EHI_148460), which were also acquired by the *E. histolytica* genome through LGT from bacterial genomes, are of unknown function. Another three proteins containing LIM zinc finger domains (EHI_022960, EHI_110280 and EHI_194520), several kinases and small GTPAses are expected to have signalling actions through the cytoskeleton. In addition, EHI_107280 encodes a V-type ATPase from V0 complex regulating cytoplasm *p*H, which is a homologue of EHI_078250 vacuolar protein sorting protein 26 of the amoebic retromer-like complex [59]. The Ubiquitin fusion degradation protein 1 (EHI_125920), which interacts with the nuclear localization protein 4 (Npl4 in yeast and EHI_026450 in *E. histolytica*) forms a cofactor for AAA-family ATPase CDC48 for proteasome-dependent processing of ubiquitinated ER-associated proteins [60,61]; in addition, Npl4 is involved in the transport of polyadenylated RNA from the nucleus to the cytoplasm [62]. A zinc finger protein EHI_008770 presents homology to Tristetraprolin, which binds to AU-rich elements in mRNA, leading to the removal of the poly(A) tail from the mRNA and increasing rates of mRNA decay [63]. A 5′-3′ exonuclease EHI_080270 has homology to Xrn1 exonuclease, the enzyme catalysing cytoplasmic mRNA degradation in decay pathways [64]. In summary, these 86 NAT-associated genes encode factors involved in metabolic processes, biosynthesis and turnover of RNA, as well as in vacuolar traffic and protein degradation by the proteasome that are common to all four species of *Entamoeba*.

### 3.7. Expression of NATs in Syntenic Blocks

The assembly and gene annotation of *Entamoeba* genomes are largely incomplete, complicating comparisons of the transcriptomes between species. To investigate the conservation of NAT between species, we examined the expression of NAT in syntenic blocks based on the syntenic annotations in AmoebaDB. In the core-genome (*n* = 3565), 3496 and 926 genes from *E. histolytica* are syntenic with at least a second species and with all four species, respectively (Table S3, Sheet 3). For the coreNATcore (*n* = 531), 524 are syntenic between at least two species and 141 genes are syntenic in all the four species (Table S9, Sheet 1). These 141 syntenic genes are distributed among 79 contigs of the *E. histolytica* genome (Table S9, Sheet 2), with 26 contigs containing two or more genes targeted by NAT and 53 contigs contain only one NAT targeted gene (Table S9, Sheet 3). In this visual contig inspection, we did not find any correlation between the proportion of gene associated with NATs, nor with their physical mapping or the number of genes on the contigs (Supplementary Figure S3). We then applied the analysis to orthologous genes (syntenic or not) presenting NATs in *E. histolytica* (1774 genes, Table S9, Sheet 5). These are in 315 contigs, 53 of which carry only one NAT-targeted gene (Table S9, Sheet 6). The largest contig (DS57114 of 530624 bp carrying 268 genes) was then scanned. We found 74 NAT-targeted genes which are distributed along the contig; therefore, they do not form clusters. Individually located genes are numerous (*n* = 21) and non-overlapping pairs are also observed. The last map according to their forward or reverse transcription sense and positioned in a convergent, opposite or similar direction (Supplementary Figure S4). Overall, the data indicating that there is no evident systematic link between physical association of genes and NAT transcription in linear genomes.

### 3.8. Differential Gene Expression Profile of Entamoeba Histolytica Antisense RNA Transcription

**Figure 6:**
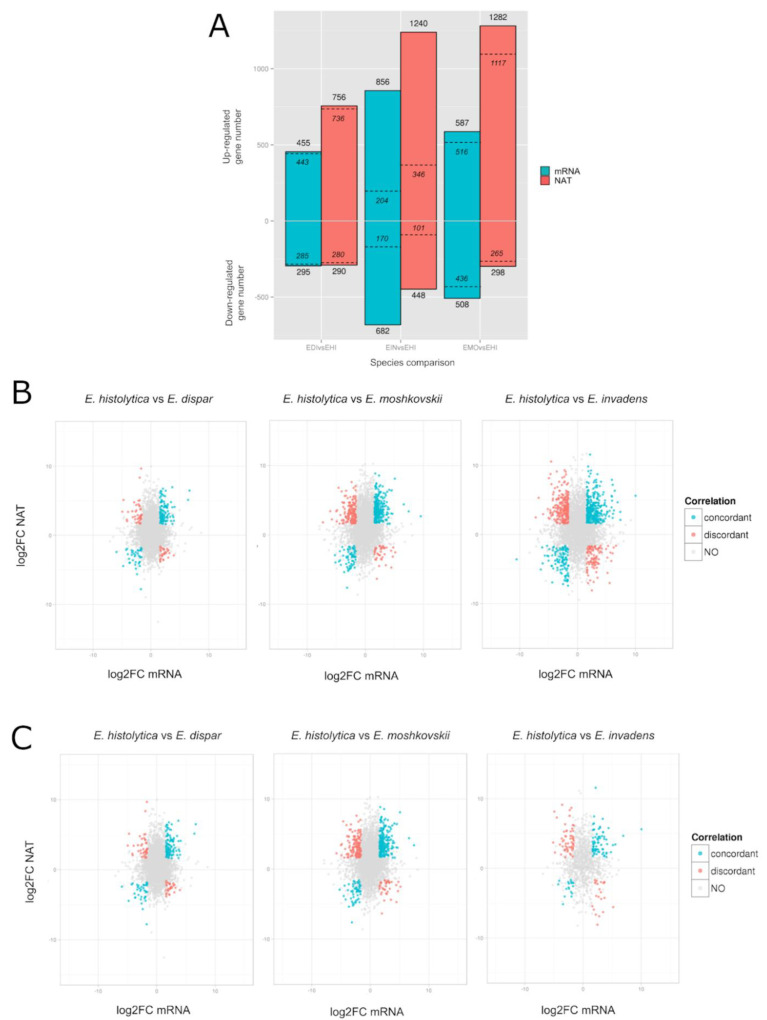
Differential expression of *E. histolytica* genes in comparison with the three other species. (**A**) Number of up-regulated and down-regulated mRNA and NATs, detailed in Table S10. Dashed lines represent the corresponding number of regulated genes in synteny between the two species. (**B**) The fold change in gene expression (FC) was compared by plotting the log2FC of mRNA (x axis) versus log2FC of NAT (y axis) for each gene having at least one NAT contig identified, for each comparison. The colour of points illustrates the differential expression type: none or unidirectional (grey), both concordant (blue), both discordant (red). (**C**) Same illustration keeping only genes in synteny between each pair of species.

We have previously profiled the gene expression changes in *E. histolytica* upon environmental change, including parasite invasion of the human colon [65]. In line with our interest in the discovery of amoebic factors related to intestinal infections, we took advantage of interspecies transcriptomic data generated in this study (three replicates from each species) to identify transcriptomic signatures specific to *E. histolytica.* Performing differential expression analyses on the core-genome (see Methods), we identified significantly differentially expressed *E. histolytica* mRNAs or NATs (fold changes > 3, fdr < 0.05) compared to another species (Figure 6 and Table S10). Among the three species, *E. invadens* showed the largest extent of differential expression, with 1544 mRNAs (856 up-regulated and 682 down-regulated) and 1688 NATs (1240 up-regulated and 448 down-regulated) differentially expressed. *E. moshkovskii* showed a moderate extent of differential expression, with 1095 mRNAs (587 up-regulated and 508 down-regulated) and 1580 NATs (1282 up-regulated and 298 down-regulated) differentially expressed. *E. dispar*, which is phylogenetically closest to *E. histolytica* [19], showed the smallest extent of differential expression, with 750 mRNAs (455 up-regulated and 295 down-regulated) and 1046 NATs (756 up-regulated and 290 down-regulated).

We also explored whether the inter-species differential expression of *E. histolytica* mRNAs is related to NATs. Interestingly, we found the up-regulated, but not the down-regulated, *E. histolytica* mRNAs are significantly associated with the presence of NATs (χ^2^ tests, *p* < 0.05, odds ratio > 1, Table S11A–C in all three species). To further investigate this phenomenon, we examined the associations between the mRNA differential expression and NATs differential expression. Surprisingly, we found significant associations (χ^2^ tests, *p* < 0.05, odds ratio > 1, Table S11D–H in all three species), independent of the direction of differential expression (i.e., up- or down-regulation) of the mRNAs or NATs. Further examining the fold changes of mRNAs and their corresponding NATs across species (Figure 6B), we can divide the mRNA/NAT pairs into “concordant” and “discordant”, based on the relative direction of their differential expression [66]. In general, we found more concordant mRNA/NAT pairs than discordant pairs. Specifically, we found concordant pairs that are up- (*n* = 113, 240 and 320) or down-regulated (*n* = 39, 65 and 113) for both mRNA and NATs expressions, and discordant pairs with up-regulated mRNA and down-regulated NAT (*n* = 30, 38 and 99) or down-regulated mRNA and up-regulated NAT (*n* = 41, 149 and 222). The significant associations of the inter-species differential expression mRNAs and NATs suggest the potential regulatory functions of NATs, or the potential linkage between biogenesis of NATs and mRNAs.

### 3.9. Functional Annotations of E. histolytica Syntenic Genes with Differentially Expressed NATs

Finally, we explored functional significance of *E. histolytica* NAT-associated genes with differentially expressed NATs between species. To avoid over-sophisticated interpretations, here we considered only syntenic genes. In the core-genome (*n* = 3565), there are 3450, 3108 and 911 *E. histolytica* genes are syntenic with *E. dispar*, *E. moshkovskii* and *E. invadens,* respectively (Table S3, Sheet 3). Examining the significantly differentially expressed NATs (fold changes > 3, fdr < 0.05) in these syntenic *E. histolytica* genes, *E. moshkovskii* showed the largest number with 1382 NATs (1117 up-regulated and 265 down-regulated), followed by *E. dispar* with 1016 NATs (736 up-regulated and 280 down-regulated) and then *E. invadens* with 447 NATs (346 up-regulated and 101 down-regulated) (Figure 6A and Tables S1–S14). Among these, there are 102 and 58 NATs that are up-regulated and down-regulated across all comparisons. Next, we examined the annotated functions of these genes with differentially expressed NATs based on Gene Ontology (GO) terms.

In comparison to *E. dispar*, among the 736 *E. histolytica* genes with up-regulated NATs, the significantly enriched processes are linked to lipid metabolism with 27 genes including the phosphatases necessary for the dephosphorylation of phospholipids (e.g., myotubularin); 31 genes are involved in small GTPases signal regulation, 13 genes are necessary for ubiquitin-dependent protein catabolic process, 3 genes are involved in DNA damage response, 3 genes are linked to protein export from the nucleus and 2 genes are implicated in RNA decapping including methylguanosine-cap decapping, which inactivates translation initiation and promotes 5′-to-3′ decay of mRNA (Table S12A). The 280 *E. histolytica* genes with down-regulated NATs can be annotated with 82 GO terms (Table S12B). Notably, they include proteins for cell adhesion, e.g., the Gal-GalNAc lectin subunit Igl2 (EHI_18300) and another under-described lectin (EHI_108490). DNA metabolic processes were also highly represented by several GO Terms with six genes involved in chromatin modification, DNA damage responses through DNA repair and DNA methyltransferase activity.

In comparison with *E. moshkovskii*, among the 1117 *E. histolytica* genes with up-regulated NATs, 423 of them are also up-regulated NATs in comparison with *E. dispar.* These genes shared 10 GO terms including lipid metabolism (*n* = 36) and nuclear export of proteins (*n* = 3). In addition, diverse global metabolic pathways were found (*n* = 94); as were response to stimuli (*n* = 92), where small GTPases signal pathways are represented by 29 genes, and DNA damage (*n* = 23, e.g., double-strand break repair MRE11, DNA mismatch repair protein PMS1 and ligases for DNA repair), while eight genes were involved in the metabolism of RNA (for example, the activity of aminoacyl t-RNA ligase) (Table S13A). The 265 *E. histolytica* genes with down-regulated NATs shared 20 GO terms with genes also with down-regulated NATs in comparison to *E. dispar* (Table S13B), including histone acetylation (*n* = 2) and protein dephosphorylation (*n* = 2). Specific to *E. moshkovskii* NAT down regulation there was supplemental protein dephosphorylation (*n* = 5), chromatin organization (*n* = 4), secretion by cells (*n* = 3), the transcription factor TFIID subunit (*n* = 1) and cAMP signalling involving adenylate cyclase activity (*n* = 1).

In comparison with the relatively distant *E. invadens*, the 346 *E. histolytica* genes with up-regulated NATs can be annotated with 43 GO terms (Table S14A). These include proteolysis, essentially ubiquitination and proteasome activity (*n* = 17); protein modification (*n* = 11), including endocytosis (e.g., clathrin adaptors AP2 and AP3) or protein translocation (e.g., SRPa, Vsp16, Vsp26, coatomer β, coatomer g, SR particle). Organonitrogen compound catabolic process (*n* = 10) which, in addition to ubiquitination process and proteasome degradation, also showed MTA/SHA nucleosidase, the transcription elongation factor B and RAD23 which plays a central role both in proteasomal degradation of misfolded proteins and DNA repair; two genes involved in gene silencing including the transcription regulator silent information regulator 2 (EHI_ 151300) and the NAD-dependent deacetylase (EHI_120280); two genes implicated in positive regulation of transcription: the transcription elongation factor SPT4 (EHI_148350) and the mediator of RNA polymerase II transcription subunit 7, (EHI_170300); four genes encoding factors necessary for tRNA modification including queuine tRNA-ribosyltransferase; the Elp1 subunit of the elongation protein complex, required for tRNA modification; and tRNA pseudouridine synthase. The other GO terms common to the other species includes eight GO terms shared with *E. dispar*, five shared with *E. moshkovskii* and two shared with both amoebae species containing few genes (e.g., phosphatidylinositol-mediated signalling, nucleoside catabolism, glycosyl compound catabolism). For the 101 down-regulated NATs the enrichment of GO terms identifies few genes. These are related to protein ubiquitination (*n* = 3), protein maturation (*n* = 2) and vesicular retrograde transport (*n* = 2) (Table S14B). Shared with *E. moshkovskii* is the presence of adenylate cyclase but there is no GO term uniquely in common with *E. dispar.*

**Figure 7:**
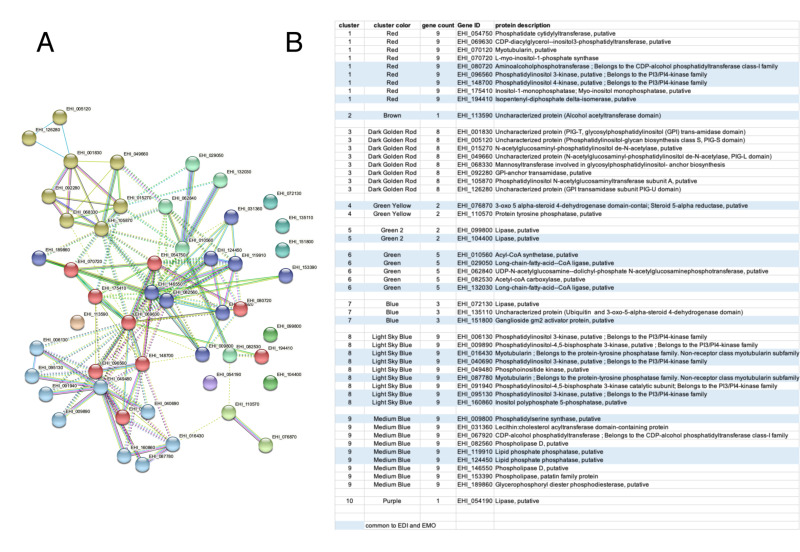
Lipids metabolism as a major target of antisense transcription in *Entamoeba* infecting humans. The test was carried out with the sum of lists of syntenic genes involved in lipid metabolism in *E. histolytica* in comparison with *E. dispar* or with *E. moshkovskii*, and which were targeted by NAT (up-regulated). A single dataset was submitted to the STRING program for gene functions clustering (K-means clustering methods). (**A**) Note the metabolism of phospholipids (red and light sky blue), biosynthesis of GPI (dark gold rod) and lipid degradation (medium blue) as the most representative among the 10 clusters analysed. (**B**) Genes found in both *E. dispar* and *E. moshkovskii* are underlined in blue, while those not underlined are the genes found in only one of these parasites.

In summary, the profile of *E. histolytica* compared to those of *E. dispar* and *E. moshkovskii* rank lipid metabolism activities as the most important enriched function specific to the species infecting humans (e.g., lipases, kinases, phosphatases and GPI biogenesis), which networking by STRING clearly distinguished the most significant gene clusters (Figure 7). Vesicular trafficking, protein degradation and RNA metabolism were found in the comparison of the four species, but regulation of RNA transcription is most pronounced in the comparison with *E. invadens*. DNA repair functions are highly specific in the comparison with *E. moshkovskii*. These findings offer new research alternatives for a better understanding of the intestinal colonization by *Entamoeba*.

## 4. Discussion

Seven species of *Entamoeba* colonize humans with different frequency; three of them are currently candidates for study because they represent more than 90% of cases of amoebic infection, namely *E. histolytica*, *E. dispar* and *E. moshkovskii*. In this work, we described and compared the transcriptomes of these three human infecting species, and a reptile infecting species, *E. invadens*, aiming to understand their phenotypic differences. All four species showed substantial levels of antisense transcription covering 40 to 59% of protein-coding genes. By genome-wide mapping of TSS and PAS, our analysis revealed the similarities and differences of the regulatory sequences determining the initiation and polyadenylation of both mRNA and NATs among the four species, expanding our previous observations [32] from an evolutionary perspective.

Analyses of NATs in the *Entamoeba* core-genome revealed a set of genes (*n* = 513) that are associated with NATs in at least two species, and are involved in cell motility, nuclear structure, cell cycle, gene transcription and transport and nucleotide metabolism, coenzymes and amino acids metabolism. Among them, 86 genes are commonly associated with NATs in all four species, representing promising loci for further studies of NAT biogenesis. Differential expression analyses of these mRNA/NAT pairs in the core-genome revealed the interdependence between mRNA and NAT expression (Table S11), implying the potential regulatory functions of NAT or the potential linkage between mRNA and NAT biogenesis.

Interestingly, a substantial number of genes involved in lipid metabolism are associated with NAT up-regulation in *E. histolytica*. These encoded enzymes involved in the degradation of lipids (lipases) and in the biogenesis of glycosylphosphatidylinositol (GPI), which is a moiety that anchors cell surface proteins to membranes, as well as kinases/phosphatases involved in the renewal of phospholipids. Multiple phospholipids play an essential role in vesicular membrane trafficking and pathogenicity of *E. histolytica* [67,68]. These results are in line with the vesicular traffic process; NAT up-regulation is also observed in genes encoding the structural components of the endoplasmic reticulum (ER) as well as small GTPase signalling factors, which are diverse, abundant in *E. histolytica* and regulate vesicular trafficking [69]. For example, small GTPases constitute signal platforms regulating endocytosis, ER protein and lipid translocation, phagocytosis and trogocytosis of human cells, as well as parasite motility.

In addition, the differential expression of NATs between *E. histolytica* and *E. moshkovskii* genes involving DNA damage and repair is particularly interesting as previous studies suggested that DNA recombination and allele reassortment should be more important than point mutations in *E. histolytica* genome [70]. In contrast, genomic divergence due to point mutations in *E. moshkovskii* strains is much greater than that demonstrated for *E. histolytica* strains [14]. One may be tempted to hypothesize that the differential expression of NATs might alter the expression of genes involving DNA damage and repair and eventually affect the frequency of DNA recombination in *Entamoeba* spp. This highly speculative consequence of NAT differential expression is expected to occur through regulating the expression of Mre11 (or other enzymes involved in DNA repair) rather than through a global mechanism dependent on antisense transcription, because the high abundance of NAT observed in this work does not correlate with the relatively low rate of recombination in *Entamoeba* species.

Although we demonstrated the existence of NAT and the interdependence of mRNA and NAT expression (Table S11) in the four species of *Entamoeba*, we are far from understanding their biogenesis and potential roles in gene regulation. In many cases, we do not observe positive correlation between NATs and mRNA expression (i.e., discordant pairs), and these discordant and concordant relationships between mRNA/NAT pairs suggest that there could be diverse mechanisms underlying their biogenesis or their potential regulatory roles in gene expression. Data from other eukaryotes indicate that NATs may regulate gene expression at the post-transcriptional or translational levels including modifying epigenetic markers [71] or specific silencing of genes [72]. In addition, antisense transcription also exists in other protozoan parasites. For example, in *Plasmodium*, NATs are associated with ~24% of coding genes, with a strong clustering towards the 3′ end of CDSs, and for some genes NAT transcript levels correlate with mRNA levels of neighbouring genes [73]. In *Toxoplasma gondii*, NATs are associated with ~21% of coding genes [74]. In *Giardia lamblia*, ~20% of sequenced cDNAs are NATs [75]. Although other examples exist [76], currently there are no clear functional roles for NATs in gene regulation in protozoan organisms. A promising recent finding shows that NAT species could represent a self-regulating mechanism of *T. gondii* gene expression at the post-transcriptional level [77]. In addition, in some fungi up to 50% of CDSs are associated with NATs [78] and it has been described that diverse phenomena such as transcriptional interference, chromatin remodelling and dsRNA formation are among the mechanisms involved in fungal gene regulation mediated by NATs [79]. Interestingly RNA interference pathways exist in *E. histolytica* and *E. invadens* [44,80]. Much remains to be learned about whether and to what extent antisense transcription in *Entamoeba* drives gene expression.

## Data Availability

The data are available in the SRA database (https://www.ncbi.nlm.nih.gov/sra/) under accession number PRJNA781395.

## Supplementary Materials

The following supporting information can be downloaded at: https://www.mdpi.com/article/10.3390/microorganisms10020396/s1, Figure S1: Small RNA linked to genes in the different species of *Entamoeba*, Figure S2: Boxplots illustrating the comparison between the specific 86 NAT associated genes and the other genes in each species (Wilcoxon-test results are bracketed), Figure S3: Genome mapping of NATs targeting syntenic genes common to the four species of *Entamoeba*, Figure S4: Mapping of NAT-targeted genes in three DNA fragments of *E. histolytica* contig DS571145, Table S1: Datasets, Table S2: Transcriptome assemblies, Table S3: Gene lists, Table S4: sRNA genes and squared-chi tests, Table S5: TSS, Table S6: PAS, Table S7: EggNOG categories, Table S8: GO enrichment, Table S9: NAT and genome contigs, Table S10: Interspecies differential expression, Table S11: Differential expression squared-chi tests, Table S12A: GO enrichment *E. histolytica* vs. *E. dispar* up-regulated, Table S12B: GO enrichment *E. histolytica* vs. *E. dispar* down-regulated, Table S13A: GO enrichment *E. histolytica* vs. *E. moshkovskii* upregulated, Table S13B: GO enrichment *E. histolytica* vs. *E. moshkovskii* down regulated, Table S14A: GO enrichment *E. histolytica* vs. *E. invadens* upregulated, Table S14B: GO enrichment *E. histolytica* vs. *E. invadens* down regulated.
