# Supplementary material for: RNA Sequencing Reveals Widespread Transcription of Natural Antisense RNAs in Entamoeba Species
Source: Microorganisms. 2022 Feb 8;10(2):396. doi: 10.3390/microorganisms10020396 (PMC8874941; doi:10.3390/microorganisms10020396)
Supplement: Supplementary file 1 [file microorganisms-10-00396-s001.zip › Supplemental Figures.pdf]

Supplemental Figures. Mornico et al., 2022

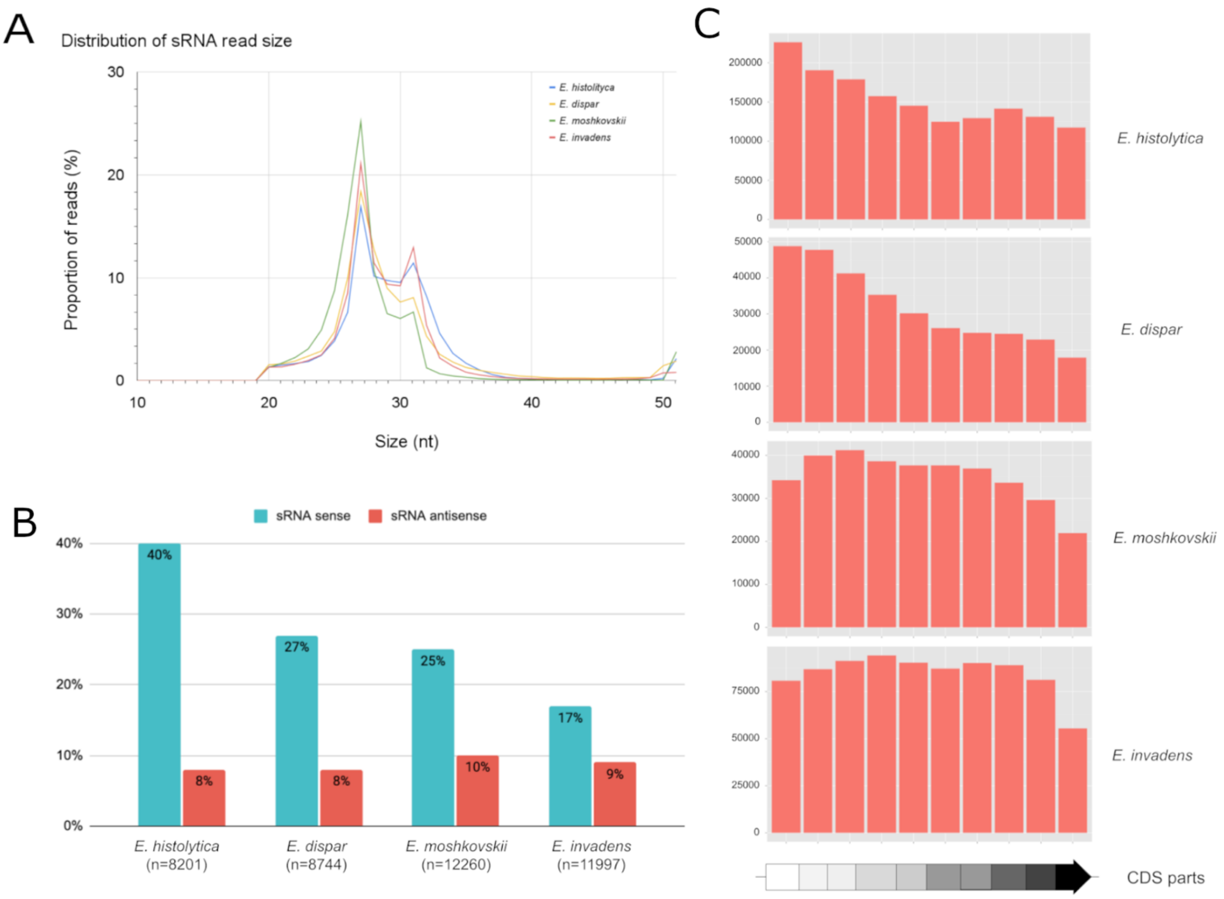

Supplemental Figure S1. small RNA linked to genes in the different species of *Entamoebae*. (**A**) Histogram of read length distribution in the small RNA libraries (**B**) Proportion of genes overlapped with sense (blue) and antisense (red) small RNA in the four species. (**C**) Mapping pattern of antisense small RNAs along the genes split in ten equal regions.

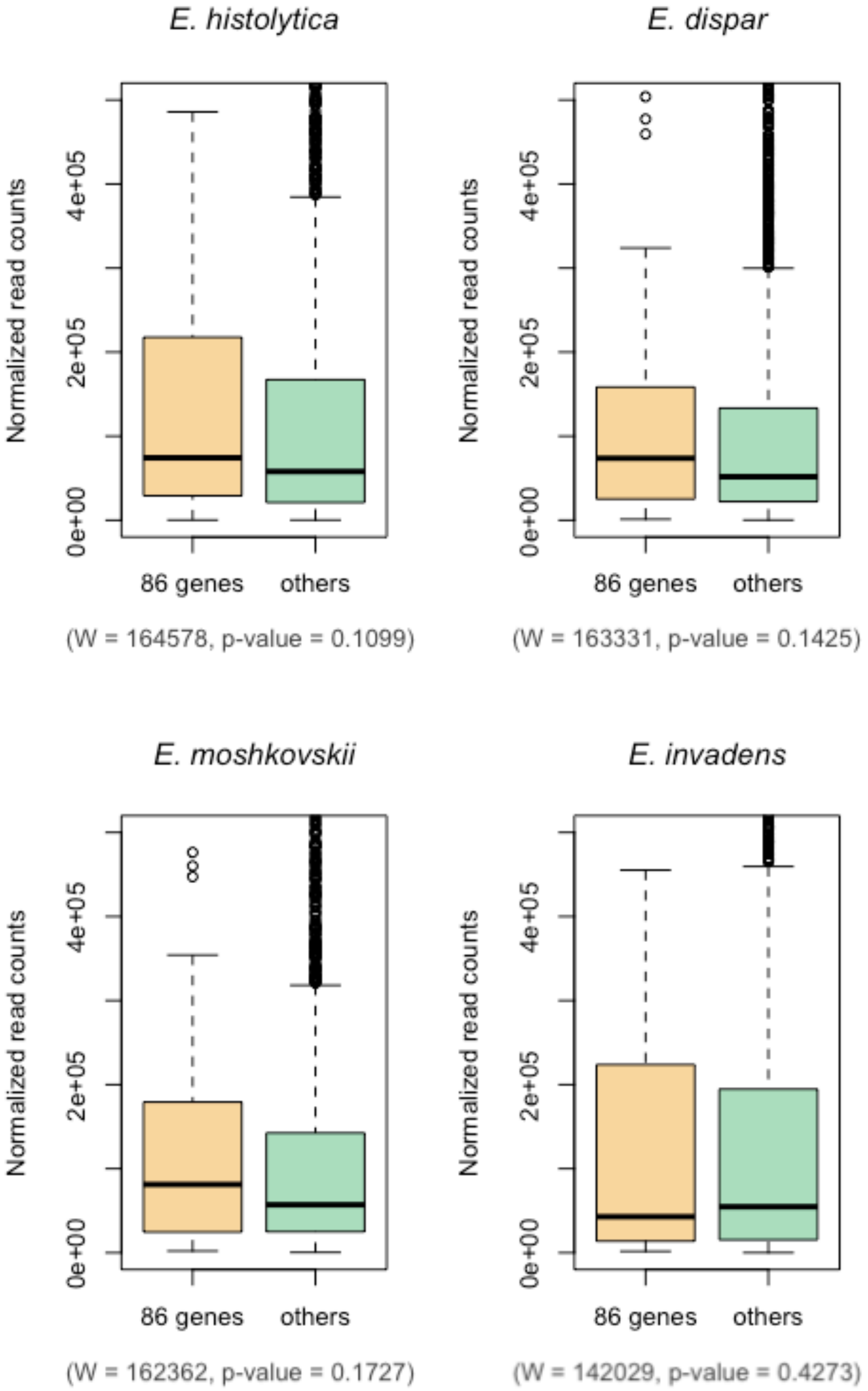

Supplemental Figure S2. Boxplots of read counts (TPM) distributions to compare gene expression of the 86 core-NAT-core genes to the others in each species. Wilcoxon-test results are indicated in brackets.

A

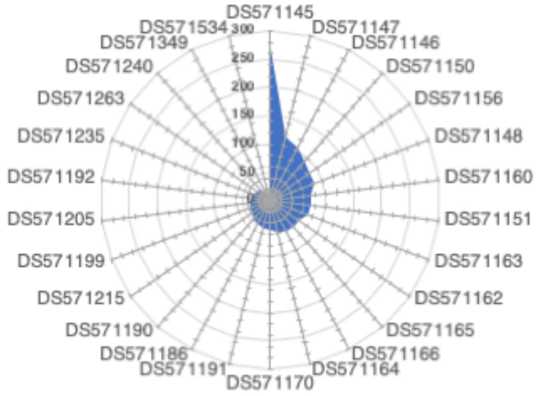

B

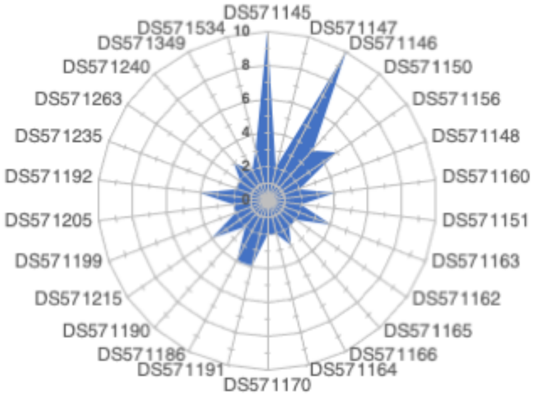

C

CONTIG	NATs	GENEs
DS571145	10	268
DS571147	2	115
DS571146	10	105
DS571150	4	93
DS571156	5	85
DS571148	2	85
DS571160	4	74
DS571151	2	73
DS571163	4	71
DS571162	2	64
DS571165	2	64
DS571166	3	62
DS571164	2	59
DS571170	2	52
DS571191	4	51
DS571186	4	47
DS571190	2	45
DS571215	4	39
DS571199	2	39
DS571205	2	34
DS571192	4	33
DS571235	2	32
DS571263	2	27
DS571240	3	25
DS571349	2	16
DS571534	3	8

Supplemental Figure S 3. Genome mapping of NATs targeting syntenic genes common to the four species of *Entamoebae.* The analysis corresponds to 141 genes of *E. histolytica* (syntenic with other species) present in coreNATcore identified in AmoebaDB database. The 26 contigs containing two or more genes targeted by NAT were considered. (**A**) Distribution of the number of genes contained in these 26 contigs. (**B**) Distribution of corresponding NATs. (**C**) Number of genes or NATs in each contig. Although the distribution of NATs is enriched for some contigs, there is no systematic proportion of NAT compared to the number of genes within the contigs.

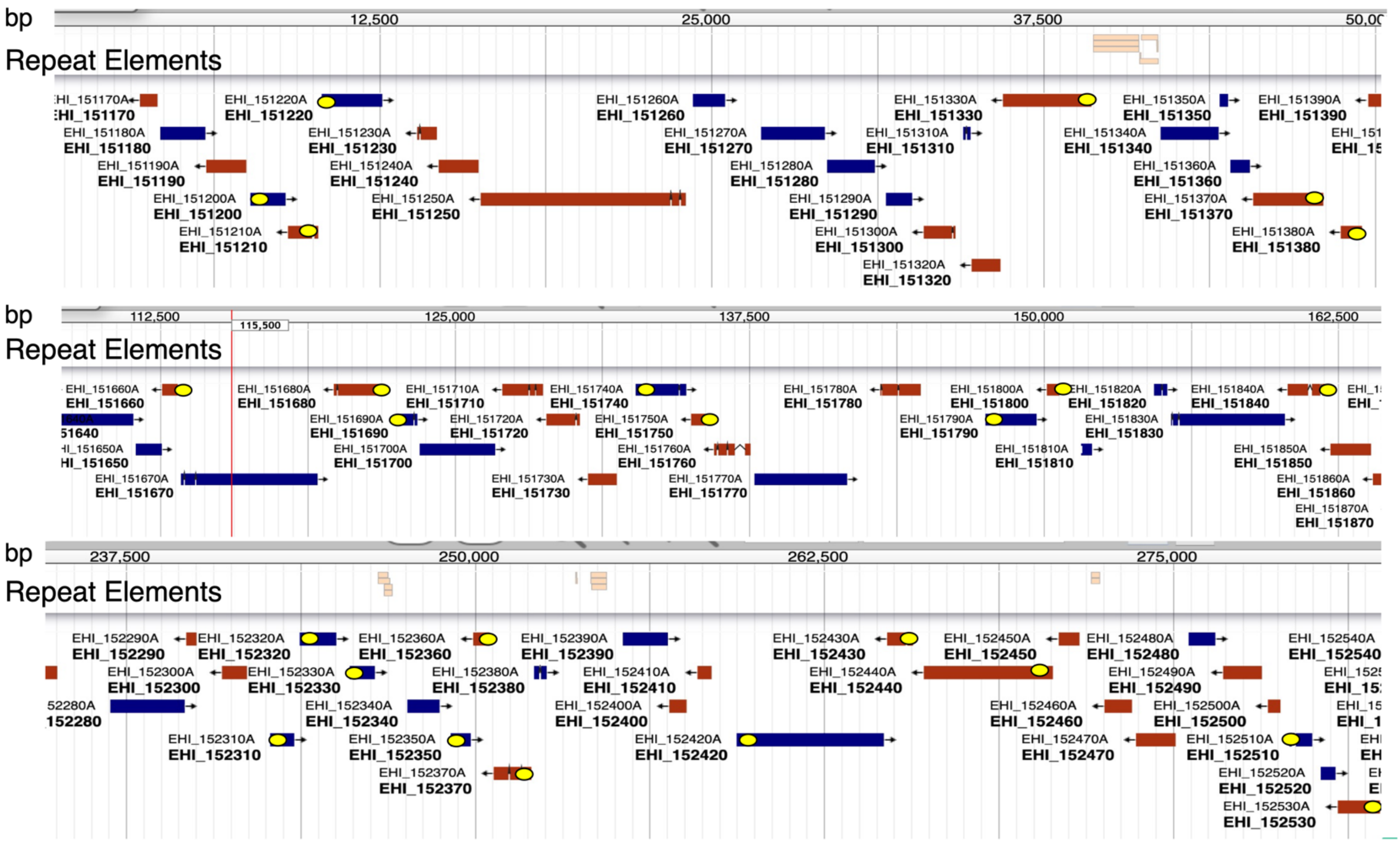

Supplemental Figure S4. Mapping of NAT-targeted genes in three DNA fragments of *E. histolytica* contig DS571145

The list of 74 genes with NAT in contig DS571145 was taken from Table S8, sheet 7. A search in AmoebaBD database (gene IDs and genomic sequences IDs) maps each gene on contigs. After visual inspection of each NAT-targeted gene in the View Genome Browser, three representative DNA fragments are displayed (top, middle, or bottom panels). Blue blocks correspond to forward-transcribed genes, red blocks correspond to reverse-transcribed genes, and the yellow dot indicates genes with NATs. Note several spatial organizations of NAT genes: individual genes, pairs of almost adjacent genes transcribed either forward or backward, these can further be arranged in convergent or divergent head-tail configuration. In some cases, pairs of NAT genes are forward or reverse transcribed. NATs do not localize with repetitive DNA elements (indicated in pink at the top of the panels); bp = base pairs.
